# Difficult Cases

**Published:** 1888-02-25

**Authors:** 


### THE NURSING MIRROR.

Communications for this column should be addressed The Mirror, care of the Editor, 38, Old Jewry, London, E.C.

### DIFFICULT CASES.

IN THE HOSPITAL for to-day, in the column headed "The Nursing Mirror," I have noticed a paragraph, "Difficult Cases." May I ask if any application has been made to Miss Macpherson's Home of Industry, Bethnal Green Road? At her Training Home at London Fields, Hackney, she receives a great many children during the winter, to be sent to Canada in the spring. Her sister, Mrs. Merry, receives them in a home there, and they are afterwards adopted into private families, or situations are found for them. Knowing how well most of Miss Macpherson's large families turn out, and having seen something of the training at Hackney, it struck me that her home would be the best place for the little girl mentioned. (Nurse) EDITH BUTCHER.

Queen Charlotte's Hospital, Marylebone-road, N.W., February 11th.

IN reply to your correspondent "Y," I beg to state that I think the "Sisters of the Poor," St. Michael's Hospital, Shoreditch, might be able to assist her in the case mentioned in her letter to THE HOSPITAL. If Miss Wilson has not written to the secretary of the Waif and Stray Society, 32, Charing Cross, about the child mentioned in her note, I should advise her to do so. That society takes children of the tenderest age. Sometimes they are only a few months old. H. THORNHILL ROXBY.

Emblewood, Osbaldeston Road, N.

